# Association between pulse wave velocity and hot flashes/sweats in middle-aged women

**DOI:** 10.1038/s41598-017-13395-z

**Published:** 2017-10-23

**Authors:** Ruwei Yang, Yang Zhou, Changbin Li, Minfang Tao

**Affiliations:** Department of Obstetrics and Gynecology, Shanghai Jiao Tong University Affiliated Sixth People’s Hospital, Shanghai, China

## Abstract

As women age and go through menopause, they suffer a higher incidence of cardiovascular morbidity and mortality. Previous studies have shown that a relationship exists between hot flashes/sweats and an increased risk of cardiovascular disease. However, the association between hot flashes/sweats and arterial stiffness is unclear. We aim to explore the relationship between hot flashes/sweats and arterial stiffness using the modified Kupperman index (KMI) questionnaire and measure the brachial-ankle pulse wave velocity (baPWV). The prevalence of hot flashes in our research was reported to be 41.77%. There was a statistically significant difference between the mean baPWV among groups that experienced different severities of hot flashes/sweats according to one-way ANOVA test (p < 0.001). The baPWV values were positively associated with the severity of hot flashes/sweats based on linear regression after adjusting for established cardiovascular confounders (95% CI: (5.86, 43.23), p = 0.01). To the best of our knowledge, this study is the first investigation to propose that baPWV may serve both as an objective index for evaluating the severity of hot flashes/sweats and as a predictor of arterial stiffness beyond Cardiac Vascular Disease (CVD) risk factors in middle-aged women.

## Introduction

Vasomotor symptoms (63.5%), fatigue (50.7%) and palpitations (35.1%) are common symptoms in peri- and postmenopausal Chinese women^1^. Hot flashes or night sweats, which are vasomotor symptoms, are regarded as thermoregulatory events that might be associated with estrogen deprivation or fluctuation^2^ and may exert a negative impact on the quality of life of menopausal women^3^. In addition, a growing body of evidence suggests a link between vasomotor symptoms and cardiovascular risks^4–6^; however, not all studies in the field agree^7–9^, possibly due to the use of different biomarkers, sample selection bias, and use of observational versus interventional studies.

The pulse wave velocity is the wave velocity between two points of an arterial system; it is positively correlated with arteriosclerosis and is useful in clinical applications to evaluate arterial stiffness^10^. The ability to measure brachial-ankle pulse wave velocity (baPWV) is commonly available in clinical practice; it is precise, not time-consuming, simple and non-invasive^11^.

Women’s risk of cardiovascular disease may increase as they progress through menopause^12,13^. Studies of the association between hot flashes/sweats and cardiovascular disease incidence reported conflicting results. It is not clear whether an association exists between hot flashes/sweats and baPWV in middle-aged women. Therefore, we aim to evaluate the impact of hot flashes/sweats on arterial stiffness by studying the correlation between hot flashes/sweats and baPWV in an effort to guide protocols for handling hot flashes/sweats and to aid in monitoring arterial stiffness in peri- and postmenopausal women.

## Results

### Participants

A total of 589 subjects were enrolled in this study; the characteristics of the study population are listed in Table 1. The mean age (SD) of participants was 50.5(5.7). The mean height, weight, BMI, systolic pressure, diastolic pressure and heart rate all followed normal distributions, the incidence of hot flashes/sweats was 41.77%.

**Table 1:** Characteristics of the study population.

Characteristic	Menopausal Status				
	Total	Pre-menopause	Peri-menopause	Early Post-menopause	Late Post-menopause
	n = 589	n = 194	n = 114	n = 140	n = 141
Age(year)	50.50 ± 5.70	45.07 ± 3.28	49.14 ± 3.60	52.95 ± 3.41	56.62 ± 3.66
Height (cm)	159.60 ± 5.03	160.44 ± 4.35	159.11 ± 4.85	159.67 ± 4.75	158.78 ± 6.07
Weight(kg)	59.56 ± 7.99	59.21 ± 7.64	59.46 ± 7.60	60.72 ± 8.11	58.97 ± 8.59
BMI(kg/m^2^)	23.38 ± 3.01	23.00 ± 2.83	23.50 ± 3.01	23.81 ± 3.02	23.38 ± 3.20
SBP(mmHg)	125.27 ± 17.87	119.93 ± 16.18	124.19 ± 19.11	127.05 ± 18.05	131.72 ± 16.65
DBP(mmHg)	76.38 ± 11.54	73.06 ± 10.07	75.64 ± 12.41	78.26 ± 10.04	79.69 ± 10.90
Heart rate(bpm)	69.81 ± 10.28	70.29 ± 9.77	68.33 ± 9.69	69.54 ± 10.07	70.60 ± 11.51
Age groups, n(%)					
40–44	131(22.2)	107(55.2)	19(16.7)	3(2.1)	2(1.4)
45–49	131(22.2)	71(36.6)	37(32.5)	18(12.9)	5(3.5)
50–54	181(30.7)	16(8.2)	56(49.1)	80(57.1)	29(20.6)
55–60	146(24.8)	0(0.0)	2(1.8)	39(27.9)	105(74.5)
Marital status, n(%)					
Married	555(94.2)	181(93.3)	110(96.5)	133(95.0)	131(92.9)
Divorced	20(3.4)	8(4.1)	2(1.8)	6(4.3)	4(2.8)
Separated, Widowed	10(1.7)	2(1.0)	2(1.8)	1(0.7)	5(3.5)
Unmarried	4(0.7)	3(1.5)	0(0.0)	0(0.0)	1(0.7)
Employment status, n(%)					
Yes	248(42.1)	125(64.4)	62(54.4)	44(31.4)	17(12.1)
No	341(57.9)	69(35.6)	52(45.6)	96(68.6)	124(87.9)
Education, n(%)					
None	52(8.8)	12(6.2)	9(7.9)	11(7.9)	20(14.2)
Primary	75(12.7)	24(12.4)	15(13.2)	21(15.0)	15(10.6)
Junior high	148(25.1)	45(23.2)	40(35.1)	30(21.4)	33(23.4)
Senior high	172(29.3)	51(26.3)	28(24.6)	46(32.9)	47(33.3)
College	130(22.1)	58(29.9)	20(17.5)	29(20.7)	23(16.3)
Postgraduate	12(2.0)	4(2.1)	2(1.8)	3(2.1)	3(2.1)
Gynecological diseases, n(%)					
Uterine fibroid	212(36.0)	56(28.9)	46(40.4)	60(42.9)	50(35.5)
Endometriosis	9(1.5)	3(1.5)	1(0.9)	4(2.9)	1(0.7)
Chronic disease, n(%)					
Hypertension	105(17.8)	23(11.9)	14(12.3)	30(21.4)	38(27.0)
Diabetes	42(7.1)	9(4.6)	4(3.5)	17(12.1)	12(8.5)
Hotflashes/sweats(yes), n(%)	41(21.1)	57(50)	61(56.4)	4(48.9)	246(41.4)

### BaPWV data

According to one-way ANOVA, there was a statistically significant difference in baPWV values across age groups (p < 0.001), menopausal status (p < 0.001), and severity of hot flashes/sweats (p < 0.001). The baPWV of each group was significantly different. (p < 0.001) (Table 2).

**Table 2:** Comparison of baPWV among different groups by age, menopausal status and degree of severity of hot flashes/sweats.

characteristics	n	mean ± SD	95% CI	
			lower	upper
Age 40–44	113	1193.32 ± 169.72*	1161.69	1224.96
Age 45–49	141	1241.54 ± 167.50*	1213.69	1269.43
Age 50–54	183	1381.92 ± 236.69*	1347.40	1416.44
Age 55–60	152	1462.64 ± 272.34*	1419.00	1506.29
Premenopause	194	1226.89 ± 185.55*	1200.61	1253.16
Perimenopause	114	1292.51 ± 188.83*	1257.23	1327.91
Early Postmenopause	140	1389.36 ± 233.12*	1350.40	1428.31
Late Postmenopause	141	1455.62 ± 289.89*	1407.35	1503.89
Hot flashes/sweats 0 points	343	1287.09 ± 227.30*	1262.95	1311.23
Hot flashes/sweats 1 point	178	1376.07 ± 229.59*	1342.11	1410.03
Hot flashes/sweats 2 points	55	1433.86 ± 289.78*	1355.53	1512.20
Hot flashes/sweats 3 points	13	1526.27 ± 243.72*	1327.84	1724.70

*Means p <0.001 CI: Confidence Interval.

Pearson’s correlation analyses revealed that systolic blood pressure, diastolic blood pressure, age, heart rate, triglyceride (TG), body mass index (BMI), and low density lipoprotein (LDL) levels were positively correlated with baPWV (p < 0.05). Height and HDL levels were significantly negatively correlated with baPWV (Table 3). Spearman’s correlation analyses revealed that hot flashes/sweats (r = 0.243, p < 0.001), menopausal status (r = 0.367, p < 0.001), and the KMI total score (r = 0.237, p < 0.001) were significantly positively correlated with baPWV menopausal status and hot flashes/sweats (Table 3).

**Table 3:** Correlation analysis of baPWV with some characteristics.

Items	r	p value
Age(year)	0.439**	<0.001
HDL(mmol/L)	−0.093*	0.024
LDL(mmol/L)	0.136**	0.001
TG(mmol/L)	0.200**	<0.001
TC(mmol/L)	0.139**	0.001
Height(cm)	−0.114**	0.006
Weight(kg)	0.081	0.050
BMI(kg/m^2^)	0.140**	0.001
Systolic pressure(mmHg)	0.594**	<0.001
Diastolic pressure(mmHg)	0.548**	<0.001
Heart rate(bpm)	0.302**	<0.001
Menopausal status	0.367**	<0.001
Flashes/sweats(points)	0.243**	<0.001
KMI total score(points)	0.237**	<0.001

**Means p <0.001; *Means p <0.05. HDL, high-density lipoprotein; LDL, Low density lipoprotein; TC, total cholesterol; TG, Triglyceride; BMI, body mass index. Hot flash and KMI total score were analyzed by Spearman’s correlation, others were computed by Pearson’s correlation.

As shown in Table 4, we observed a significant positive association between baPWV and hot flashes/sweats (p = 0.010). BaPWV was also significantly associated with SBP, age, hypertension, heart rate, and diabetes mellitus in the full multiple linear regression model following corrections for age, BMI, HDL, LDL, TC, TG, DBP, height, and menopausal status.

**Table 4:** Linear regression analysis of baPWV with hot flashes/sweats in an adjusted model.

Traits	Unstandardized coefficients		95% CI	p value	Corrected R²
	B	Std Error			
SBP(mmHg)	3.76	0.64	(2.5, 5.025)	<0.001	0.54
Age(year)	10.67	1.31	(8.11, 12.24)	<0.001	
Hypertension(yes)	−147.53	20.21	(187.21, 107.84)	<0.001	
Heart rate (bpm)	3.68	0.70	(2.31, 5.05)	<0.001	
Diabetes mellitus(yes)	−98.58	27.04	(−151.69, −45.46)	<0.001	
Flashes/sweats	24.54	9.52	(5.86, 43.23)	0.010	

Covariates: BMI, HDL, LDL, TC, TG, DBP, height, menopausal status.

## Discussions

Based on 589 women’s modified KMI scores, the prevalence of hot flashes/sweats was found to be 41.77%, which is similar to previous findings from other Asian countries, such as Japan, Hong Kong, Singapore^14^ and South Korea^15^, and was significantly lower than that found in white populations^16,17^. The discrepancy may be explained by the racial and cultural context^18^, demographic and socioeconomic characteristics^19^, and methods of symptom identification^20^.

This study revealed that the pulse wave velocity is significantly associated with aging, menopausal status and duration of menopause; this finding parallels the relationship between the decrease in estrogen levels in menopausal women and vascular aging. As a result, baPWV is considered to be a possibly effective measure to evaluate arterial stiffness in middle-aged women.

Our study found that the pulse wave velocity was positively correlated with the frequency of hot flashes/sweats and the severity of symptoms (r = 0.243, p < 0.001). After adjusting for established cardiovascular risk factors, such as systolic blood pressure, diastolic blood pressure, age, menopause, heart rate, TG, BMI, TC, LDL, HDL, and hot flashes/sweats (95% CI: 5.86–43.23, p = 0.01), the results remain significant using a linear regression analysis. Therefore, we can safely infer that the assessment of baPWV is a valuable tool to evaluate symptoms of hot flashes/sweats, the method provides an objective standard by which to assess symptoms of hot flashes in menopausal women.

It is known that SBP, DBP, age, menopause, BMI, TG, TC and LDL are risk factors for cardiovascular diseases^21^. Our analyses have shown that SBP, DBP, age, heart rate, TG, BMI, TC, LDL were significantly positively correlated with baPWV, which is consistent with previous studies^22^. After adjusting for these items in addition to a history of hypertension and diabetes mellitus, the independent risk factors for higher arterial stiffness were found to be age, systolic blood pressure, history of hypertension and diabetes mellitus, heart rate and hot flashes/sweats. We can therefore extrapolate self-reporting hot flashes/sweats has clinical implications for predicting arterial stiffness in menopause beyond other CVD risk factors.

Additionally, we should pay more attention to hot flashes/sweats. We suggest that people who experience severe hot flashes/sweats should focus on atherosclerosis and baPWV, and health staff should prioritize the treatment of people with hot flashes/sweats by, for example, providing menopausal hormone therapy.

To the best of our knowledge, this may be the first study to suggest that hot flashes/sweats and their severity are associated with higher baPWV. We suggest that baPWV may serve as a metric to monitor arterial stiffness for middle-aged women. Additionally, baPWV can likely be regarded as an objective index for evaluating the severity of hot flashes/sweats; furthermore, self-reporting hot flashes/sweats is of prominent value to assess arterial stiffness independent of CVD risk factors in middle-aged women. however, it is worthy of further exploration and research. Because of the limited survey samples, more community-based studies on large groups of people are important. Our team will continue to conduct future relevant research to confirm the validity of these results. We are now conducting a cohort study of measuring baPWV before and after MHT to explore changes in pulse wave velocity after MHT in middle-aged women.

### Limitations

Several limitations need to be mentioned. First, the inherent drawback of an observational survey may attenuate the causal relationship. Secondly, systemic errors due to the baPWV tool may be produced, for its calculation of path length comes from a height-based formula for Japanese population. However, the height of Chinese is similar to that of Japanese. Finally, hot flashes/sweat ascertained by questionnaire would produce memory bias. Therefore, further longitudinal study is needed to confirm these relationships. Our team is now working on the following-up investigation.

## Conclusions

1. BaPWV may serve as a metric to monitor arterial stiffness in middle-aged women
2. BaPWV may provide an objective standard to evaluate the severity of vasomotor symptoms.
3. Self-reporting hot flashes/sweats may be regarded as an easy and quick predictor of arterial stiffness independent of CVD risk factors in middle-aged women.

## Methods

### Study Subjects

Women who received a physical examination at the Center of Health Examination of Shanghai Jiao Tong University Affiliated Sixth People’s Hospital from the 28st of November 2016 to the 28th of September 2017 were enrolled in this study. The inclusion criteria included the following: 1) aged between 40 and 60 years; 2) normal cognition, has the ability to complete the questionnaire by herself; and 3) volunteered to participate in this research. The exclusion criteria included the following: 1) female who received menopausal hormone treatment (MHT) or any traditional Chinese medicine indicated for menopause in the past 6 months; 2) history of mental disorder; and 3) history of serious organic diseases (e.g., coronary heart disease, stroke, systemic autoimmune disease).

A total of 589 women met to the criteria. The reproductive medicine center of the Shanghai Sixth People’s Hospital institutional review board approved this study, This analytic cross-sectional study enrolled 1904 participants aged 40–60 years who visited the physical examination center in the Shanghai Sixth people’s Hospital, Shanghai Jiao Tong University School of Medicine, China, from January 2016 to November 2016. The study protocol was approved by the Ethics Committee of Shanghai Sixth People’s Hospital, and the study was performed in accordance with the approved guidelines. All the participants provided written informed consents after full explanation of the study.

### Questionnaire

The women’s health demographic questionnaire included basic information, such as age, education, marital status, occupation, history of MHT and traditional Chinese medicine, menopausal states, menstrual states, gynecological history, and past history. This questionnaire has been applied previously^23,24^.

### Menopausal status group

According to the stages of reproductive aging workshop (STRAW + 10)^25^, we divided subjects into the following groups: premenopausal (with regular menstrual cycle), perimenopausal (consecutive irregularities >7 days from their normal cycle), early postmenopausal (absence of menstrual periods for at least 12 months and less than 5 years) and late postmenopausal (absence of menstrual periods for more than 5 years).

### Menopausal symptoms and hot flashes/sweats group

We used a valid modified KMI scale in Chinese to evaluate the severity of menopausal symptoms^26^. The modified KMI scale consists of three aspects: 1) somatic symptoms, such as hot flashes/sweats, palpitation, vertigo, headache, paresthesia, formication, arthralgia and myalgia; 2) mental symptoms, such as fatigue, nervousness and melancholia; and 3) genitourinary tract symptoms, such as urinary infections and sexual complaints. The sum of the scores from 0 to 63 points were categorized into four grades:

1. score ≤6, asymptomatic (148 subjects)
2. 6< score ≤15, mildly symptomatic (259 subjects)
3. 15< score ≤30, moderately symptomatic (159 subjects)
4. score >30, severely symptomatic (23 subjects)

According to the modified KMI subproject, we divided the hot flashes/sweats group into four groups:

1. 10 point, no hot flashes/sweats (342 subjects)
2. 1 point, hot flashes and sweats ≤3 times/day (178 subjects)
3. 2 points, 3< hot flashes and sweats ≤9 times/day (55 subjects)
4. 3 points, hot flashes and sweats >9 times/day (14 subjects).

### Measurement of PWV

We used an automatic waveform analyzer (BP-203RPE III, OMRON, Japan) to measure baPWV. Participants were asked to remain supine and at rest for 5 minutes before the PWV examination^27^. We record the heart rate concurrently.

### Laboratory examination

All participants underwent a fasting lipid profile consisting of total cholesterol (TC), triglycerides triglyceride (TG), high-density lipoprotein (HDL)and low density lipoprotein (LDL). Height, weight and systolic blood pressure (SBP), diastolic blood pressure (DBP) were determined on the same day. Body mass index (BMI) was computed by dividing weight in kilograms by the square of their height in meters.

### Statistical analysis

SPSS Statistics 23.0 (IBM Corporation, Armonk, NY, USA) was used for all analyses. All variables were presented as the mean ± standard deviation (SD) or number (%). One-way ANOVAs were used to analyze the variation in PWV by menopausal status and degree of hot flashes/sweats. Pearson correlation analysis was performed to evaluate the relationship between age, HDL, LDL, TC, TG, height, weight, BMI, SBP, DBP, heart rate and baPWV. Spearman correlation analysis was performed to evaluate the relationship between menopausal status, hot flashes/sweats and baPWV. Associations with the pulse wave velocity and hot flashes/sweats were computed with a linear regression analysis. R² values were derived from linear regression models. Residuals analysis was performed and diagnostic plots were made to verify model assumptions. A two-sided P-value < 0.05 was considered statistically significant.
